# BGEM: An In Situ Hybridization Database of Gene Expression in the Embryonic and Adult Mouse Nervous System

**DOI:** 10.1371/journal.pbio.0040086

**Published:** 2006-03-28

**Authors:** Susan Magdaleno, Patricia Jensen, Craig L Brumwell, Anna Seal, Karen Lehman, Andrew Asbury, Tony Cheung, Tommie Cornelius, Diana M Batten, Christopher Eden, Shannon M Norland, Dennis S Rice, Nilesh Dosooye, Sundeep Shakya, Perdeep Mehta, Tom Curran

## Abstract

This article describes an open-access gene expression database analyzed for more than 2,000 genes on mouse nervous system tissue in the coronal, sagittal, and transverse orientation representing multiple developmental ages.

## Introduction

The challenge of the postgenomic era is not only to assign functions to individual genes, but also to determine how sets of genes act in concert to control biological processes. This formidable task is even more daunting when one attempts to understand the complex genetic programs underlying nervous system development. More than half of the approximately 25,000 genes in the mouse genome are thought to be involved in development and function of the nervous system [ 1, 2], but only 30% of genes have any function assigned to them [ 3]. Identifying the temporal and spatial expression patterns of these genes throughout development is a critical initial step that lays the groundwork for additional functional analyses. Toward this goal, we have developed a publicly available database of gene expression patterns, the St. Jude Brain Gene Expression Map (BGEM).

BGEM ( http://www.stjudebgem.org) is a growing collection of in situ hybridization images of gene expression patterns in the nervous system of the developing and adult C57BL/6 mouse. Data are displayed on an image-centric Web site in a format that enables easy visualization of temporal and spatial changes in gene expression. Currently, the information in BGEM is used to select candidate genes for use in constructing BAC transgenic mice as part of the Gene Expression Nervous System Atlas (GENSAT) project ( http://www.ncbi.nlm.nih.gov/entrez/query.fcgi?db=gensat and http://www.gensat.org). The GENSAT project is designed to document the expression patterns of all genes in the nervous system and to generate transgenic mice expressing reporter constructs that recapitulate the authentic expression patterns of selected genes. GENSAT is supported by the National Institutes of Neurological Disorders and Stroke and by the National Institutes of Health Neurosciences Blueprint ( http://neuroscienceblueprint.nih.gov), a partnership of 14 NIH institutes and centers committed to accelerating understanding of the nervous system.

## BGEM Database

**Figure 1:**
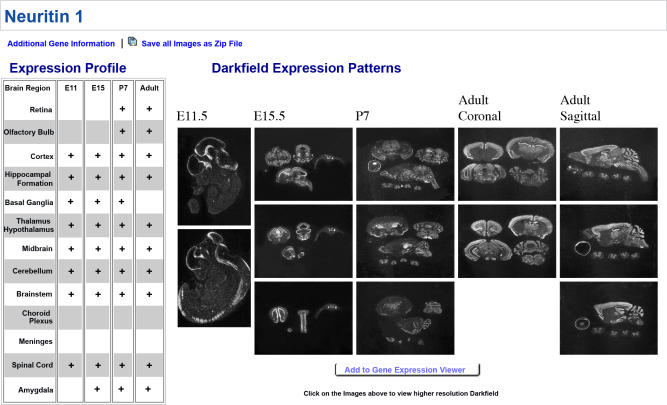
Thumbnail Images Displayed in BGEM Showing Expression Data for *neuritin1*, *(Nrn1)* This arrangement allows simultaneous visualization of gene expression patterns in early embryonic development (E11.5 and E15.5), postnatal (P7), and adult nervous system.

The BGEM database contains a survey of gene expression patterns at four critical stages of mouse nervous system development: embryonic day 11.5 (E11.5), E15.5, postnatal day 7 (P7), and adult (P42). Using optimized high-throughput radioactive in situ hybridization techniques and a novel tissue-blocking system, each probe is hybridized to at least 54 individual tissue sections (detailed methods are available at http://www.stjudebgem.org). Darkfield images of each probe are displayed initially as a set of thumbnails to provide a snapshot of temporal and spatial gene expression patterns throughout development ( Figure 1). Each thumbnail image is linked to an intermediate-sized image for convenient comparison with a nearby Nissl-stained reference section, and this is linked to the original full-sized image that can be downloaded. BGEM provides a side-by-side “gene expression viewer,” allowing assembly of custom collections of gene expression patterns for further analysis. The collections can be modified over time and shared with other users or printed as a “contact sheet” to facilitate comparisons. The gene expression viewer has proven to be an ideal tool for comparing gene family members, genes whose products participate in ligand–receptor interactions, and genes involved in signal transduction pathways. BGEM can be browsed, or the database can be systematically searched using a variety of gene identifiers such as the official gene symbol, name, alias, genetic location, and/or gene ontology terms. The “bulk search” feature can be used to upload lists of genes, making large searches possible. Biological information from search results can be readily exported to an Excel document, allowing systematic comparisons with other databases or information sets.

For each gene, we provide up-to-date information available from NCBI ( http://www.ncbi.nlm.nih.gov). BGEM is also linked to PubMed, UniGene, Entrez Gene, and the Gene Ontology Consortium ( http://www.geneontology.org). Additionally, gene information can be obtained through links with the Online Mendelian Inheritance in Man (OMIM) Web site ( http://www.ncbi.nlm.nih.gov/entrez/query.fcgi?db=omim), the GENSAT databases at Rockefeller University ( http://www.gensat.org) and NCBI ( http://www.ncbi.nlm.nih.gov/entrez/query.fcgi?db=gensat), the Mutant Mouse Regional Resource Centers ( http://www.mmrrc.org), the Microarray Consortium ( http://arrayconsortium.tgen.org/np2/home.do), and the Mouse Genome Informatics database ( http://www.informatics.jax.org).

## Genes in the BGEM Database

BGEM contains more than 30,000 images, representing expression data obtained from more than 2,400 unique probes hybridized to more than 129,000 individual sections of nervous system tissue. The 2,400 unique probes correspond to more than 12,000 gene ontology terms and categories. For example, there are 224 genes from the G-protein-coupled receptor protein signaling pathway, and 375 genes have either kinase or phosphatase activity. In addition, more than 600 genes are either receptors or associate closely with receptors, providing a thorough analysis of important signaling pathways. BGEM contains images for more than 400 genes with DNA binding and/or transcription factor activity, and more than 300 genes with protein translation and protein transport activity. Thus, the BGEM database provides users with expression information for a broad range of genes in an easy-to-use online format that will complement a great variety of basic research investigations.

The genes in BGEM were selected in part by groups of neuroscientists with expertise in neurodevelopment, neurodegeneration, receptors and channels, and cognitive neuroscience. In addition, several cDNA clone collections, including the National Institute of Aging 15K mouse clone collection, the Incyte 1.0 Unique mouse clones, and the Brain Molecular Anatomy Project collection [ 4, 5], were queried to identify cDNAs derived from nervous system tissues or those involved in biological processes such as neurogenesis, migration, differentiation, cell proliferation, cell death, and metabolism. Microarray studies of brain tissues and the characterization of genes associated with human neurological disorders (OMIM) were also used to select genes for BGEM. These selection strategies proved fruitful in identifying genes with patterns that change during development or that highlight discrete populations of positive cells. The expression patterns of 65% of the genes in BGEM are either temporally restricted, spatially restricted, or both during brain development. A key feature of the high-throughput process coupled to the selection strategy is that equal weight is given to both characterized and uncharacterized genes. This equal-weighting feature has uncovered several interesting expression patterns ( Figures S1–S3). Examples include predicted genes revealed by genome sequencing [ 6] and RIKEN-derived genes [ 7] ( Figures S1A, S1G, S2B, and S3F). Thus, the data provided in BGEM represent a first step in the characterization of these novel genes. For example, we have identified gene expression patterns within the nervous system for many genes, including *heat-shock 70-kDa protein 12A (Hspa12a), keratocan (Kera),* and *dermatan sulfate proteoglycan 3 (Dspg3),* that had previously been reported in only non-neuronal tissues ( Figures S1I, S2C, and S2F). In addition, BGEM includes well-characterized genes such as *SLIT-ROBO Rho GTPase activating protein (Srgap1)* ( Figures S1F and S2G), *solute carrier 17, member a6 (Slc17a6)* ( Figures S2A and S3E), *neuro-oncological ventral antigen 1 (Nova1)* ( Figure S2D), and *D0H4S114* ( Figures S1F and S2G, S2A and S3E, S2D, and S2E, respectively). In most cases, the previously published expression data comprise only a single age or tissue or were generated by non-histological molecular biology techniques. BGEM contains a more extensive, growing in situ collection of developmental gene expression data. Thus, it is important to complete our goal of characterizing the expression patterns of all genes in the mouse genome to provide a comprehensive picture of the complexities of gene regulation in the nervous system.

## Comparison with Other Gene Expression Databases

BGEM differs in several ways from the two other major online gene expression databases, GenePaint ( http://www.genepaint.org) and the Allen Brain Atlas ( http://www.brainatlas.org), that provide images of neuronal tissues analyzed by in situ hybridization. BGEM is the only database that utilizes the radioactive detection method, whereas GenePaint and the Allen Brain Atlas utilize non-radioactive probes. One advantage of radioactive probes is that they provide greater sensitivity for genes expressed at lower levels and they have a better signal-to-noise ratio than non-radioactive probes. In addition, it is much easier to discern gradients of gene expression in darkfield images of radioactive probes than in the color images of non-radioactive probes. Most of the genes in GenePaint show expression patterns for only E14.5 embryos in the sagittal plane, whereas BGEM displays at least three developmental ages, ranging from E11.5 to P42, in two to three planes of orientation for every gene entry. The Allen Brain Atlas contains images from only adult mouse brain, so there is no information on developmental expression patterns. The Mahoney transcription factor database ( http://mahoney.chip.org) is not currently being annotated or updated. Therefore, BGEM provides the most comprehensive gene expression analysis of any of the major databases.

Currently, there are fewer than 350 genes, representing less than 2% of the estimated number of protein-coding genes in the mouse genome, that are present in both the BGEM and GenePaint databases. There is greater gene overlap between the adult gene expression data in the Allen Brain Atlas and BGEM. We find that the three Web sites are quite complementary, and it is very useful to compare the same gene in BGEM with one of the other atlases. This way, it is possible to examine a broad collection of images that, taken together, provide adequate sensitivity and resolution for most purposes. Ideally, it would be best if there were direct links among genes from all of the databases. However, for this to occur all databases would need to be published in an open-access format.

## Conclusions

Linking multiple databases in an open-access information network will open up unprecedented opportunities for scientific information exchange. Already, we see great benefit from the reciprocal links between gene expression patterns in BGEM with the Mutant Mouse Regional Resource Centers and with GENSAT. Users can view gene expression patterns in BGEM and compare them with high-resolution images from GENSAT, and they can request BAC reporter mice from the Mutant Mouse Regional Resource Centers directly through the linked Web sites. Gene expression data acquired through other methodologies, such as transcriptome analysis of nervous system tissue, could also be linked to BGEM, enhancing the utility of both datasets.

The electronic integration of multiple experimental disciplines will establish an invaluable resource that will accelerate investigations of nervous system development and disease. For example, BGEM allows users to combine gene expression information with data from other experimental studies. Gene-chip studies often produce an overabundance of candidate genes upregulated or downregulated in a single experiment. Knowing the temporal and spatial context of gene expression in the developing nervous system can provide key information for categorizing these results and pinpointing the most important genes for further analysis. Recently, BGEM was used to complement a gene expression microarray approach to the etiology of human brain tumors. Taylor et al. used microarray analysis to define molecularly distinct subsets of human ependymomas that arise in different brain regions [ 8]. The developmental gene expression patterns of 71 signature genes were examined by mining the BGEM database. The authors found that tumors arising from specific anatomical locations maintained expression of genes that were present in similar locations during development. This led to the hypothesis that ependymoma subgroups were derived from distinct populations of radial glia cells. This strategy illustrates the utility and power of BGEM, which can link several genomic technologies in a systems biology approach. The essential components necessary for high-throughput in situ hybridization analysis described here are based on cost-effective routine laboratory practices that do not require robotics. The methods can be readily adapted for high-throughput analysis of gene expression in any tissue or model organism, and they may also be expanded to accommodate complementary technologies such as immunohistochemistry, mass spectrometry, or other imaging modalities. Current efforts in the neuroscience community to standardize anatomical territories and nomenclature will provide an avenue for information exchange and cross-indexing of experimental protocols, gene expression annotations, and variations in tissue preparations [ 9, 10]. Since BGEM was created using a MySQL platform, direct links with other databases are possible. The BGEM database could be emulated by others to make large datasets conveniently interoperable. We look forward to linking our image sets to other gene expression databases that are actively growing.

The data in BGEM are available without restriction. BGEM images may be incorporated into grant proposals, scientific presentations, and manuscripts by citing the BGEM URL ( http://www.stjudebgem.org) and this publication. BGEM is a free resource that represents a new avenue for information exchange that will accelerate understanding of the nervous system.

## Supporting Information

Figure S1 Examples of E11.5 Gene Expression Patterns in BGEM Darkfield images are displayed for the following genes: (A) *hypothetical LOC553091, LOC553091*, (B) *neuro-oncological ventral antigen 2, Nova2,* (C) *protocadherin 9, Pcdh9*, (D) *endothelin receptor type B, Ednrb*, (E) *WAP four-disulfide core domain 2, Wfdc2*, (F) *SLIT-ROBO Rho GTPase activating protein 1, Srgap1*, (G) *BAC clone RP24-69C19 from Chromosome 6*, (H) *early B-cell factor 2, Ebf2*, and (I) *heat shock 70kDa protein 12A, Hspa12a*. All images were obtained from the highest-resolution images available at http://www.stjudebgem.org.
(3.5 MB TIF).

Figure S2 Examples of P7 Gene Expression Patterns in BGEM Darkfield images are displayed for the following genes: (A) solute carrier family 17, member 6, Slc17a6, (B) similar to transcriptional repressor scratch 2, LOC545474, (C) keratocan, Kera, (D) neuro-oncological ventral antigen1, Nova1, (E) DNA segment, human D4S114, D0H4S114, (F) dermatan sulfate proteoglycan 3, Dspg3, and (G) SLIT-ROBO RhoGTPase activating protein 1, Srgap1. All images were obtained from the highest-resolution images available at http://www.stjudebgem.org.
(3.4 MB TIF).

Figure S3 Examples of Adult P42 Gene Expression Patterns in BGEM Darkfield images are displayed for the following genes: (A) cholecystokinin, Cck, (B) complexin 1, Cplx1, (C) G protein-coupled receptor 6, Gpr6, (D) interferon activated gene 203, Ifi 203, (E) solute carrier family 17, member 6, Slc17a6, (F) RIKEN cDNA 6430573F11, 6430573F11Rik, and (G) S100 calcium binding protein A10 (calpactin), S100a10. All images were obtained from the highest-resolution images available at http://www. stjudebgem.org.
(3.2 MB TIF).

**Accession Numbers**

The NCBI ( http://www.ncbi.nlm.nih.gov) accession numbers for the genes and gene products discussed in this article are *6430573F11Rik* (NM_176952), *BAC clone RP24-69C19 from Chromosome 6* (AC124500), *Cck* (NM_031161), *Cplx1* (NM_007756), *D0H4S114* (NM_053078), *Dspg3* (NM_ 007884), *Ebf2* (NM_010095), *Ednrb* (NM_007904), *Gpr6* (NM_199058), *Hspa12a* (NM_175199), *Ifi 203* (NM_008328), *Kera* (NM_008438), *LOC545474* (XM_ 619828), *LOC553091* (BC052230), *Nova1* (XM_356586), *Nova2* (NM_001029877), *Nrn1* (NM_153529), *Pcdh9* (XM_139187), *S100a10* (NM_009112), *Slc17a6* (NM_ 080853), *Srgap1* (XM_905068), and *Wfdc2* (NM_026323.

## Abbreviations

BGEM: St. Jude Brain Gene Expression Map
E[number]: embryonic day [number]
GENSAT: Gene Expression Nervous System Atlas
P[number]: postnatal day [number]
